# Illness perception in pediatric somatization and asthma: complaints and health locus of control beliefs

**DOI:** 10.1186/1753-2000-1-5

**Published:** 2007-07-16

**Authors:** Lutz Goldbeck, Silke Bundschuh

**Affiliations:** 1 Department of Child and Adolescent Psychiatry/Psychotherapy, University Hospital Ulm, Steinhoevelstr. 5, D-89075 Ulm, Germany; 2 Department of Pediatrics, University Hospital Ulm, Prittwitzstr. 43, D-89075 Ulm, Germany

## Abstract

**Background:**

Health- and illness-related cognitions of pediatric patients with asthma or somatization and of their caregivers are considered relevant for patient education and for cognitive-behavioral interventions. This study investigates the relationship between diagnosis and illness perception by child and parent in two different chronic conditions such as somatization disorder and asthma.

**Methods:**

25 patients with somatoform disorders and 25 patients with asthma bronchiale completed the Giessen Complaint List and the Multidimensional Health Locus of Control Scale. Primary caregivers independently answered parallel proxy-report instruments. Analyses of variance were performed to determine the impact of diagnosis and perspective. Correlations were calculated to determine the concordance between patient and caregiver reports.

**Results:**

No statistically significant differences in illness locus of control beliefs were found between asthma and somatoform disorder children or parents. Parents reported more internal and fatalistic locus of control beliefs compared with their children. Correlations between patient and caregiver reports of symptoms and health locus of control beliefs were low to moderate.

**Conclusion:**

Clinicians should take into account a sense of insufficient symptom control in both diagnostic groups and different viewpoints of patients and their parents.

## Background

Somatoform disorders and asthma bronchiale are two of the most frequent chronic conditions in childhood and adolescence with prevalence rates between 2.7 per cent [1] and 13.1 per cent for somatization including pain disorder [2] and between 4.25 per cent [3] and 9.3 per cent [4] for asthma. In both conditions cognitive-behavioral interventions aim to change patients' and their caregivers' maladaptive perceptions and enhance adaptive cognitive strategies [5]. The effectiveness of cognitive-behavioral treatments has been demonstrated for somatoform disorder such as recurrent abdominal pain in children [6] and for the improvement of adherence to treatment regimens in children and adolescents with asthma [7]. Thus, the usefulness of addressing the patients' cognitions seems to be evident. However, only few studies have investigated illness concepts in pediatric patients and their caregivers. Therefore our knowledge of the patients' illness-related cognitions is limited.

The patients' perception and evaluation of somatic symptoms are considered important for subsequent illness behavior and coping with the disease. Somatoform disorders are characterized by a specific way of exaggerated attention to and negative evaluation of bodily sensations [8,9]. Whereas patients who somatize are considered over-sensitive regarding their bodily functions [10], patients with asthma bronchiale need to monitor their pulmonary function and perceive early symptoms of dyspnea to utilize medical treatments before the onset of severe states of asthma [11].

The locus of control construct refers to the subjective beliefs of control that patients have over illness and health. Applying Rotter's social learning theory [12] to patients with chronic diseases, three different styles of health- and illness-related locus of control cognitions have been described, representing illness experiences and generalized expectancies of symptom control [13]. *Internal locus of control *represents the belief that one's own behavior is regarded as important for one's state of health. This attitude is regarded as an essential pre-condition for active coping strategies both in patients with somatoform disorder and asthma. *Social externality *means that powerful others, for example parents or physicians, are considered as important for symptom control. This concept triggers the help-seeking behavior of patients. Patients with *fatalistic health locus of control *beliefs are convinced that their health state is influenced by fate, luck or random events. Fatalistic expectations are considered maladaptive in both somatoform disorder and asthma because of the associated passive patient behaviour [14].

In pediatric patients, the responsibility of parents for the illness behavior of their children and the caregivers' impact on the development of the children's own subjective health concepts have to be considered. Parents monitor their children's health state, decide whether medical care is to be sought and comply with medical recommendations or not. The children's and adolescent's illness-related perceptions may be influenced by parental models and suggestions [15,16]. On the other hand, different stages of cognitive development are known to determine subjective illness concepts [17,18], thus systematic differences between children and adults are proposed. So far there are few studies on the relationship between parents' and children's illness concepts. Perrin and Shapiro [13] reported absent correlations between parental and children's health locus of control perceptions. Such different viewpoints of parents and their children might complicate intervention planning.

To our knowledge, so far no studies have compared the health locus of control beliefs of children and adolescents with somatoform disorder and with asthma bronchiale, and the corresponding parental perceptions and cognitions. The comparison of subjective illness representations would contribute to answer the question whether disease-specific cognitions have to be addressed in cognitive behavioral interventions. Moreover, the comparison of patients' and their caregivers' perceptions would allow differential planning of family-oriented psychoeducational interventions. In this exploratory study, two research questions are addressed:

1) Do patients and their parents develop disease-specific health- and illness-related locus of control beliefs? As medical treatment in combination with the patients' active role in symptom control is effective for asthma but not for somatoform disorder, we would expect more social external and internal health locus of control beliefs in the asthma patients and their caregivers, compared with patients with somatoform disorder and their caregivers.

2) Are parental proxy-reports and patients' self-reports on somatic complaints and locus of control beliefs correlated? In accordance with previous findings in pediatric recurrent abdominal pain [19] and several other chronic conditions [13], we hypothesized a weak or absent association between children's and parental symptom reports and illness concepts.

## Method

### Design and procedures

Informed consent of caregivers and assent of children and adolescents to participate in the study were obtained in accordance with the principles of the local ethical committee. The assessment was done by a researcher independently from the physicians and therapists responsible for the patients' treatment. All participants filled in standardized questionnaires, the caregivers provided additional information about individual illness history, and socio-demographic data. Patients and caregivers filled in the questionnaires independently from one another.

### Sample

Patients between 8 and 18 years of age with a clinical diagnosis of somatoform disorder or asthma bronchiale and their primary caregivers were included (for details see table 1). The study sample represented a consecutive number of patients who were eligible for the study at the participating study centres within a certain time frame. Mothers were the most frequent parental responders (86%) in both clinical groups. Younger children and mentally retarded patients were excluded, because they were not able to read and answer the questionnaires. Twenty-five children and adolescents with different subtypes of somatoform disorders and their primary caregivers were approached at specialized psychosomatic outpatient units of two university clinics. The spectrum of diagnoses included broadband somatization symptoms, autonomous dysfunction, and pain disorders. Diagnoses had been established by clinical psychologists or psychiatrists according to ICD-10 F45.x diagnostic criteria (equals DSM IV 300.81 somatization disorder or undifferentiated somatoform disorder; 307.80 chronic pain disorder associated with psychological factors; or 307.89 chronic pain disorder associated with both psychological and a general medical condition). Twenty-five inpatients with asthma bronchiale (ICD-10 J45.x) and their caregivers were recruited within the first few days after admission to a rehabilitation centre for pediatric pulmonology. The study participants with asthma represented rather a selection of non-responders to treatment within the system of primary healthcare. No significant between-group differences appeared at a level of *p *< .05 in socio-demographic variables such as age, gender, family constellation, socio-economic status or school absence. All patients were Caucasians.

**Table 1:** Medical and socio-demographic characteristics of study sample

*variable*	*psychosomatic patients (n = 25)*	*patients with asthma bronchiale (n = 25)*
specific diagnoses (ICD-10/DSM-IV)	2 somatization disorder (F45.0/300.81)	25 asthma bronchiale (J45.x/n.a.)
	4 undiff. somatization disorder (F45.1/300.81)	
	6 somatoform autonomous dysfunction (F45.3/300.81)	
	13 persistent pain disorder (F45.4/300.80 or 300.89)	
duration of disease	5 <6 months	(1 no information)
	13 6–24 months	4 6–24 months
	7 >24 months	20 >24 months
patients' age (years)	mean = 12.8, SD = 2.9, range 8–18	mean = 11.4, SD = 2.6, range 8–16
patients' gender	18 female, 7 male	13 female, 12 male
absence from school (days/year)	mean = 23.0, SD = 29.7, range 0–130	mean = 10.3, SD = 18.7, range 0–80
socio-economic status (vocation)	7 low, 17 medium, 1 high	4 low, 21 medium
family constellation	21 complete	17 complete
	2 stepfamilies	2 stepfamilies
	2 single parent families	4 single parent families

### Measures

Subjective complaints were assessed with the *Giessen Complaint List for Children and Adolescents (GBB-KJ *[20]). This multi-dimensional self-report questionnaire contains a broad range of different subjective somatic complaints. The child or adolescent is asked to report the frequency of each complaint on a 5-point rating scale (0 = never, 1 = rarely, 2 = sometimes, 3 = often, 4 = permanent). A parallel parent-form measures proxy-perceptions of their child's symptoms. Five subscales with seven items each measure the dimensions *fatigue, gastrointestinal symptoms, limb pain, circulation symptoms*, and *symptoms of a cold*. A total score indicating severity/diversity of complaints is calculated by summing the raw scores of the five subscales. Good reliability and validity has been reported previously [21]. In the present study, internal consistency scores were between *α *= .74 and *α *= .85 for the subscales and .89 respectively .90 for the global complaint scale.

The *Multidimensional Health Locus of Control Scale (German version: KKG *[22]) measures three dimensions of perceived controllability of individual health and illness symptoms: *internal *health locus of control, *social external *locus of control, and *fatalistic external *locus of control. Each scale consists of seven items with different perceptions of controllability of health- and illness-related aspects. On 6-point rating scales (1 = do not agree to 6 = agree very strongly) the degree of assent to each statement has to be indicated by the respondent. The questionnaire has been developed and validated with different clinical and healthy groups of children, adolescents and adults. It has good reliability and validity. In our study, the internal consistency was *α *= .68 for the socio-external scale, .85 for the fatalistic-external scale, and .87 for the internal dimension.

In order to receive additional parental reports, we modified the *KKG *for the perspective of caregivers by rewording the items, for example "If my son/daughter has complaints, we ask somebody for advice" instead of "If I have complaints, I ask somebody for advice." Cronbach's *α *for the parent form was .66 for the socio-external scale, .72 for the fatalistic external scale and .79 for the internal locus of control dimension.

### Statistical analyses

A descriptive analysis of the different scores was performed separately for both clinical groups and both informants. There were no statistically significant differences in socio-demographic and clinical characteristics between the two diagnostic groups. A series of 2 × 2 ANOVARs was computed with diagnosis (somatoform disorder, asthma) as between-subject factor and different dimensions of self-reported and caregiver-reported locus of control beliefs as repeated measures within-subject variables. *Pearson *correlations between self-reports and parental proxy-reports in corresponding scales of the questionnaires were calculated to determine inter-rater concordance. We also calculated intra-class correlations, and the resulting scores were approximately the same as indicated by the Pearson correlations. To avoid redundant information, only Pearson coefficients are reported. A significance level of α = .05 was chosen. With regard to the explorative character of the study and to reduce the risk of *β*-errors, no adjustment of significance level for multiple testing was made.

## Results

### Complaints

A broad variance of different complaints was found within both clinical groups, as reported by patients and caregivers in the *Giessen Complaint List for Children and Adolescents (GBB-KJ*[20]). On the level of syndrome scales, self-reported *fatigue *and *gastrointestinal symptoms *in the psychosomatic patients and parent-reported *cold symptoms *in the asthma group were most frequent. On the single-symptom level, self-reported and parent-reported *cough *was the most frequent symptom in the asthma group, self-reported and parent-reported *headache *and *abdominal pain *were the most frequent single symptoms in the somatization group. Psychosomatic patients and their parents reported significantly more symptoms compared with pulmonologic patients, especially in the *gastrointestinal *and *circulation *dimensions.

### Health locus of control

The most prominent manifestation of health locus of control perceptions was the parental *fatalistic *locus of control perception in the asthma group (*mean *= 33.1, *SD *= 6.0), whereas the least manifestation of health locus of control was found in the asthma patients *internal *dimension (*mean *= 21.1, *SD *= 6.5). No significant differences in health locus of control estimations were demonstrated between the diagnostic groups (see table 2). However, there were significant main effects of perspective. In the *internal *and *fatalistic locus of control *perceptions parents scored higher than patients. In the *fatalistic *control perception there was a statistical trend towards a greater advantage of the parents vs. their children in the asthma group compared with the somatization group. Across both clinical groups, the multidimensional profiles of health locus of control beliefs revealed a significant preference of *fatalistic external *health control beliefs in the parents (*mean *= 30.6, *SD *= 6.5), compared to less frequent *internal *(*mean *= 27.7, *SD *= 5.4) and even lesser *socio-external *health control beliefs (*mean *= 24.7, *SD *= 5.0; *ANOVA*: *F *= 18.9, *p *< .001). In contrast, there were similar levels of each control attribution in patients (*F *= 1.3, ns).

**Table 2:** Effects of diagnosis^1 ^and perspective^2 ^on health locus of control beliefs^3^

	*SOMATOFORM DISORDER*		ASTHMA		ANOVAR		
	
	*parents*	*patients*	*parents*	*patients*	*single effect: *	*single effect: *	*interaction effect: *
	
**Health locus of control:**	*mean (SD)*	*mean (SD)*	*mean (SD)*	*mean (SD)*	*diagnosis F (p)*	*informant F (p)*	*diagnosis × informant F (p)*
Internal	28.0 (5.5)	25.4 (7.9)	27.5 (5.5)	21.1 (6.5)	2.2 (.143)	**15.9 (< .001)**	2.4 (.124)
Socio-external	23.9 (4.7)	23.3 (5.9)	25.4 (5.2)	22.4 (6.1)	<1 (ns)	2.7 (.105)	1.0 (ns)
Fatalistic external	28.2 (6.2)	24.7 (8.1)	33.1 (6.0)	24.0 (7.2)	2.4 (.130)	**20.2 (< .001)**	4.0 (.052)

^1 ^somatoform disorder, asthma bronchiale

^2 ^parent proxy reported, patients' self report

^3 ^measured by the Multidimensional Health Locus of Control Scale;

results of ANOVARs for different diagnosis (somatoform disorder, asthma bronchiale) as independent variable and the repeatedly measured (parent proxy reported, patients' self report) health locus of control beliefs as dependent variables; *df *= 3, 47

### Associations between parental and self-report data

Correlational analyses demonstrated weak associations between patients' and parents' reports, both separately within the clinical groups and in the total study sample (see table 3). Symptom reports were correlated significantly on a low to moderate level. Parent-patient correlations appeared slightly stronger in the psychosomatic group, compared with the asthma group, especially in the circulation and gastro-intestinal symptom scores. The only significant inter-rater correlation in the health locus of control perceptions could be demonstrated for the internal dimension, due to a moderate parent-patient concordance in the psychosomatic group. Interaction effects of diagnosis and informant occurred in the complaint list *total score *and in the *cold *subscale, demonstrating a parental under-estimation of children's subjective symptoms in the somatization group and a parental over-estimation of symptoms in the asthma group.

**Table 3:** Patient-caregiver concordance (*Pearson *correlations) in corresponding complaint scores^1 ^and health locus of control dimensions^2^

	Total sample (N = 50)		Asthma (n = 25)		Somatoform disorder (n = 25)	
	
Variable	*r*	*p*	*r*	*p*	*r*	*p*
Fatigue	.27	.066	.23	ns	.29	ns
Gastro-intestinal symptoms	.46	.001	.20	ns	.55	.005
Limb pain	.43	.002	.50	.011	.41	.044
Circulation symptoms	.47	.001	.08	ns	.52	.010
Cold symptoms	.39	.005	.36	.077	.48	.018
Total complaints	.30	.040	.22	ns	.35	.092
						
Internal health locus of control	.31	.028	.10	ns	.48	.015
Socio-external locus of control	.15	ns	.09	ns	.24	ns
Fatalistic external locus of control	.00	ns	-.03	ns	.06	ns

^1 ^Giessen Complaint List

^2 ^Multidimensional Health Locus of Control Scale

## Discussion

This study investigates differential effects of diagnosis (somatoform disorder vs. asthma) on subjective illness-related cognitions and compares patients' and their caregivers' health- and illness-related symptom perceptions and locus of control evaluations.

The analysis of the locus of control beliefs demonstrate differences between parental and patients' cognitions, but not between patients or parents of different diagnostic groups. Across both diagnostic groups parents have more pronounced control beliefs than their children. This finding is significant for the internal and fatalistic health locus of control dimension, whereas only a statistical trend occurs in the difference between caregivers and patients in the socio-external control attribution.

The attribution of symptoms to fate or chance indicates a sense of lack of control on illness, and consequently high scores of fatalistic-external locus of control are rather expected correlated with passive illness behavior and non-adherence to treatment. According to our results, especially parents of children and adolescents with asthma develop high fatalistic beliefs, indicating a lack of subjective predictability of their children's asthma symptoms so far. This might be due to the long history of ineffective asthma treatment in our study sample. However, the asthma patients themselves report no significantly elevated fatalistic-external control perceptions compared with their internal or socio-external attributions of symptom control. In another study with the same instrument Schmitt et al. [14] found even a lower level of fatalistic locus of control beliefs in adolescent patients with asthma. A selection bias may be responsible for this finding, because in our study sample the patients were younger and non-response to standard treatment was a frequent reason for admission to inpatient rehabilitation.

Another relevant finding is that the asthma patients in our study sample reported low scores of perceived internal controllability of symptoms. Internal health locus of control has been demonstrated as associated with adherence to treatment [23] and should therefore be enhanced by therapists. Both results – the low internal control attribution of asthma patients and the high fatalistic control perception of their caregivers indicate the need for patient education and training.

The differences between the patients' and their caregivers' health related locus of control beliefs might also be explained by developmental differences of cognitive functioning. The belief that health cannot be controlled and that fate or chance might be responsible for staying healthy or becoming ill, requires the awareness of limited personal power. Due to their tendency towards concrete and rather egocentric thinking, children may overestimate their personal impact or the influence of powerful others on their health.

Our results demonstrate the ubiquity of subjective illness concepts across both chronic conditions. These findings are consistent with those of Perrin and Shapiro [13] who found no disease-specific control attributions of mothers with chronically ill children. Absence of disease specific locus of control beliefs may be explained with similar experiences of both clinical groups. Somatoform disorders and asthma bronchiale are both chronic conditions without prognosis of immediate cure, and both clinical subgroups had a longer history of ineffective treatment within the primary healthcare system before entering our study.

With regard to our second research question, we demonstrated that accordance between parents and patients is limited both in terms of symptom reports and cognitive illness concepts. Symptom reports were correlated only moderately. Consistent with the different diagnoses, children and adolescents with somatization disorder reported significantly more symptoms than peers suffering from asthma bronchiale. However, this judgment is not supported by the parents, who reported a similar level of symptoms in both clinical groups. Several explanations for this discrepancy between self reports and parent reports are possible. First, the ability of caregivers to recognize and report internal perceptions of their children reliably may be limited [19]. Patients may conceal some of their symptoms from their parents, and therefore parents cannot give valid reports on their child's subjective health status. Secondly, the discrepancies may be due to reporter biases. Patients who somatize may aggravate their symptoms because they need to legitimate their illness state, and their parents may be non-respondents to attention-seeking strategies of their children, which become manifest in somatic complaints [16]. Asthma patients may have successfully adapted to their disease and consequently developed a recall bias, neglecting and under-reporting the negative aspects of their disease. On the other hand, parents of asthma patients may over-estimate the severity of their children's symptoms because of a fearful monitoring, thus becoming over-sensitive for indicators of restricted physical well-being in their ill child [24]. As demonstrated previously in the study of Perrin and Shapiro [13], self-reported and parent proxy-reported health locus of control attributions were only moderately correlated in the internal dimension and not at all correlated in the external dimensions.

In summary, the results of our study demonstrate substantial differences between patients' and parental illness-related perceptions, whereas subjective illness concepts varied independently of diagnosis.

Several limitations of this study should be mentioned. First, the results have to be considered as preliminary because of its possible selection bias. The participants with psychosomatic disorder may have been altered in their health- and illness-related cognitions by impact of psychological or psychiatric diagnosis and/or treatment compared with non-referred psychosomatic patients. The participants with asthma were referred to a specialized inpatient rehabilitation program, therefore they may have been more resistant against basic patient education and counselling than average pediatric asthma patients in outpatient settings. It can be assumed that our asthma sample represents rather non-responders to treatment within primary care, so these patients might be more difficult to treat and even more altered in their health perceptions and illness beliefs. With regard to these limitations due to selection, our results cannot be generalized to all patients with somatoform disorder or asthma.

Secondly, the small sample size may have concealed small between-group differences because of a limited statistical power. Large scale studies comparing different diagnostic subgroups might be able to detect differences that did not occur in our study because of its restricted sample size.

Moreover, the findings in this study are based on standard self-report measures. The socio-external health locus of control scale suffered from sub-optimal internal consistency of α < .70 and should therefore be interpreted cautiously. Future studies should integrate semi-structured interviews and a qualitative methodology to collect more detailed information on the structure of subjective illness perceptions. Also possible mediating factors on symptom perception and illness concepts such as anxiety should be integrated in future study designs.

However, our preliminary results contribute some interesting findings to the emerging literature on symptom perception and cognitive aspects of chronic pediatric conditions. For the first time, we evaluated the health locus of control perceptions of children with Somatizationand their parents, and found similar cognitions as found in pediatric asthma. Future studies should include larger samples in different clinical settings and multi-method designs. Longitudinal studies would be necessary to get information on changes of subjective illness perceptions and cognitions.

## Conclusion

Some clinical implications emerge from our findings. Our results indicate the relevance of multi-informant strategies in the diagnosis of health- and illness-related perceptions and cognitions in pediatric patients. Collecting parallel self-reports and caregiver reports of symptoms and health locus of control attributions should be an obligatory part of diagnosis. Such a comprehensive diagnosis is useful for planning patient education and counselling. Our findings highlight the importance of exploring patients and their caregivers separately to get valid information on subjective symptoms and illness concepts. Patients themselves may be regarded as more reliable informants about somatic symptoms, whereas both the patients' and their caregivers' subjective illness concepts are relevant for targeting cognitive-behavioral interventions. The degree of patient-parent accordance is an additional relevant aspect of diagnosis and intervention planning. Clinicians have to consider different subjective viewpoints of chronically ill children and their caregivers, and cognitive-behavioral interventions have to take into account these different health and illness concepts.

## Competing interests

The author(s) declare that they have no competing interests.

## Authors' contributions

LG conceived in the study, participated in its design and coordination, performed the statistical analyses and drafted the manuscript. SB participated in the design, collected the data, and helped to analyse the data. Both authors read and approved the final version of the manuscript.
